# Metabolic Syndrome and Outcome Predictions: Friends or Foes?

**DOI:** 10.3390/jcdd12110421

**Published:** 2025-10-23

**Authors:** Alessandro Menotti, Paolo Emilio Puddu

**Affiliations:** 1 Association for Cardiac Research, 00182 Rome, Italy; amenotti2@gmail.com; 2 EA 4650, Signalisation, Électrophysiologie et Imagerie des Lésions D’ischémie Reperfusion Myocardique, Normandie Université, UNICAEN, 14000 Caen, France

**Keywords:** outcome prediction, CHD, CVD, Akaike Information Criterium, analytical treatment of risk factors, metabolic syndrome, serum cholesterol, Cox models

## Abstract

Objectives: An analysis based on epidemiological material to show whether the term Metabolic Syndrome (MS) should be adopted when aiming at predicting coronary heart disease (CHD) and major cardiovascular disease (CVD) fatal events. Material and Methods: MS was defined according to the International Diabetes Federation (IDF) and risk factors were identified in the Italian Risk Factors and Life Expectancy (RIFLE) population study covering over 25,000 adult men from a pool of 19 Italian population samples. The original MS definition and the plain original units of measured risk factors were challenged in Cox proportional hazard models predicting 196 CHD and 412 major CVD fatal events in a seven-year follow-up. Parallel models were run including also total serum cholesterol as a covariate, an unfortunately excluded covariate in the MS definition. The performance of the various models was tested by the log-likelihood statistics treated with the Akaike Information Criterium (AIC). Results: Models using the plain measurements of the risk factors involved were systematically and significantly outperforming any other categorized score based on the IDF-MS classification. An intermediate role was played by a model where the predictive variable was a factor score (derived from a Factor Analysis) where the MS risk factors were linearly combined. The same models also including serum cholesterol provided a significantly better prediction when compared with those without serum cholesterol, based on AIC. Conclusions: The use of a subset of classical CVD risk factors classified according to the IDF-MS criteria adds nothing better than the exclusive use of the risk factors treated by traditional procedures. The addition of serum cholesterol definitely helps in the prediction of the CHD component of major CVD events. Its omission is erroneous.

## 1. Introduction

The story of metabolic syndrome (MS) goes back several decades with the identification of the common association between obesity, hypertension, dyslipidemia and glucose intolerance, later called and classified as MS [1,2]. The problem aroused a lot of interest and soon several working groups created a large number of criteria to define MS, usually based on the same four components (obesity, hypertension, dyslipidemia and glucose-diabetes) but frequently adopting different sub-components, and almost always using different cut-off limits to define the presence–absence of a component or a subcomponent [3,4,5,6,7,8]. These proposals were subsequently used by practicing physicians and investigators and many published analyses claimed the value of these scored criteria for selecting cases of MS and, accordingly, predicting cardiovascular diseases (CVD) and diabetes. However, the adopted criteria were frequently thought to be ambiguous, a potentially common causality seem uncertain, there is no basis for including (or excluding) new components, and the CVD risk seems basically dependent upon the levels of the single risk factors while their a priori combination into the scored term MS apparently adds nothing. All this to say that we are facing something that was defined based on an unclear description, of uncertain origin, probably of little practical use and, for sure, a source of confusion.

To complicate the issue, subsequent proposals were made to add other components, like C-reactive protein, uric acid, homocysteine, fibrinogen, albuminuria and others. The fact is that the PUBMED publication platform has offered, during the last five quinquennia, about 100,000 papers where “Metabolic Syndrome” is called for. Despite this success, there are investigators, coming from the clinical perspective, complaining that MS is neglected [9]. The net consequence is that whatever bibliographic mention is made, it will be judged incomplete and/or irrelevant. A couple of major analyses predicting CVD in the USA and Europe [10,11] may be exemplified. In addition, MS was shown to predict almost all types of gastrointestinal cancers [12] and lung cancer [13]. By adding to MS some variables related to kidney function, the Cardio-Kidney Metabolic Syndrome [14] was more recently created. All this means that MS seems to be the universal predictor, if not cause, of all major diseases in clinics. By contrast, not a word was spent on MS, apart from the 34% prevalence among chronic obstructive pulmonary disease patients, in the 2021 European Society of Cardiology Guidelines on cardiovascular disease prevention in clinical practice, developed by the Task Force for cardiovascular disease prevention in clinical practice with representatives of the European Society of Cardiology and 12 medical societies with the special contribution of the European Association of Preventive Cardiology [15]. Clearly, this contrasts with the subjective opinion raised and expressed without quotations that “The importance of the metabolic syndrome is not in risk prediction in the first place since performance of risk prediction models based on individual components of the metabolic syndrome will be better in terms of discrimination and calibration.” [16]: clearly without tested comparisons.

Critics of the idea of MS came rather early and, almost incredibly, even from the proper initiator of the idea who set the stage for a “requiescat in pace” stated that just “being alive is not good enough” [17,18], a concept that evolved also from others [19,20]. Given this incredibly complex and confusing situation, our purpose was to make a dedicated statistical analysis wherefrom it might be objectively assessed whether there are indeed advantages to clustering several recognized CVD risk factors into a categorized MS definition. The alternative would be to follow subjective cut-off limits and ignoring and omitting many other important determinants (such as total serum cholesterol), for prediction of major CVD and of coronary heart disease (CHD) in particular and disregarding other evidence piled up by population studies during the last 60–70 years.

The purpose of this analysis was to identify if there is any real advantage to creating a “Syndrome” [namely MS] out of a sub-group of more or less recognized cardiovascular risk factors, and to classify them by a priori categorized and scored criteria, with the purpose of predictingmajor fatal CVDand CHD in particular.

## 2. Material and Methods

### 2.1. Populations

We used data from the Italian Risk Factor and Life Expectancy (RIFLE) project, a relatively old population study that assembled various cohorts examined at baseline and then followed up for a few years to collect mortality data [21]. Although the original study included 52 cohorts of men and women, this analysis was restricted only to men and to a subgroup of cohorts whose data were complete and reliable. Since the end-point was major fatal CHD and CVD events during a median follow-up of 7 years, women were excluded as they produced too few cases, despite carrying a similarly large denominator.

The baseline examination involved 25,271 cardiovascular-disease-free men aged 35–70 years (out of the original 26,331 men and after excluding the cardiovascular diseases prevalent cases) derived from 19 cohorts spread throughout 11 Italian regions.

### 2.2. Measurements

For this analysis we used age (as a major confounder) and a few risk factors employed in the classical definition of MS, i.e., body mass index (a proxy of waist circumference that was not available in the Italian RIFLE project), systolic and diastolic blood pressure, HDL cholesterol, plasma triglycerides and blood glucose. These are the risk factors used for the definition of MS according to the International Diabetes Federation (IDF) [8] rules. Although the deviation was that body mass index instead of waist circumference was used, they both stand for similar but imperfect proxies of visceral fat [16] and body mass index was considered an option to substitute waist circumference [4]. Moreover, as a part of the analysis, total serum cholesterol was added as it is not acceptable to exclude it when dealing with major CHD and CVD predictions [15]. Measurement techniques are reported elsewhere [21].

The IDF-MS (MSIDF) classification [8] was therefore used considering the following four components, all expressed as yes–no (1–0): (1) Obesity = Body mass index ≥ 30 units; (2) Hypertension = Systolic blood pressure ≥ 130 or Diastolic blood pressure ≥ 85 in mmHg; (3) Dyslipidemia = HDL cholesterol < 40 mg/dL or triglycerides ≥ 150 md/dL; and (4) blood glucose > 100 mg/dL.

The following arbitrary groupings of the IDF-MS classification were used together with the risk factors there included (analytical groups): (A) MSIDF = the presence of body mass index ≥ 30 units plus 2 or more other components defined by IDF-MS (yes–no; 1–0), since this is its official definition; (B) MSFA = the individual factor score derived from a Factor Analysis produced with the 6 risk factors used by the IDF criteria. A Factor Analysis is a statistical procedure that creates scores by combining different factors that are independent from the opinions of the investigators (thus a posteriori) and is based on pre-defined inter-correlations across the used variables; and (C) MSLINEAR = the original values of each of the single risk factors used in IDF-MS. These analytical groups of risk factors plus age, and separately with the addition of total serum cholesterol, were used as covariates in two series of Cox models described later.

As the end-point for this exercise we selected two groups of CVD (accompanied by the codes of the 9th Revision of the WHO-ICD-9) [22], [in parenthesis]: (a) a group of fatal major CVD including typical coronary heart diseases (CHD: myocardial infarction, acute ischemic attack, sudden coronary deaths) [410, 411, 412, 414 and 798 if classified as CHD], other major CVD in the absence of typical CHD (heart failure [428], chronic arrhythmia [427], blocks [426], hypertensive heart disease [402.1, 404], most of cerebrovascular diseases [430–434, 436–438] and major peripheral artery diseases [440.2, 441, 442, 443], for a total of 412 fatal events; and (b) a group limited to a selection of CHD’s most specific and typical syndromes [410, 411, part of 412, 798]. The selection of the above groups was oriented to specific groups, then excluded other choices.

Causes of death were attributed by a single trained and supervised coder following defined rules. In case of multiple causes and uncertainties about the principal cause, a rank choice was adopted with violence, cancer, CHD, stroke and other causes, in that order.

### 2.3. Statistical Analysis

Mean levels with standard deviations and medians with interquartile percentiles of the selected risk factors were computed together with the prevalence rates of the analytical groups. Risk factors distribution was tested for normality and in all cases it was rejected, but Cox models can manage covariates without a clearcut Gaussian distribution. A correlation matrix was produced across the levels of risk factors used for the analysis.

The following Cox proportional hazards models were computed with fatal major CVD and, separately, CHD, as end-points and the following covariates as predictors: (a) Age and MSIDF; (b) Age and MSFA and; (c) Age and MSLINEAR risk factors. The same models were replicated with the addition of total serum cholesterol (expressed as original measurement in mg/dL).

The performance of the various models was evaluated and compared. In particular, pairs of log-likelihood statistics were compared after the transformation into the Akaike Information Criterium (AIC) [23]. Lower levels of AIC correspond to a better accuracy of prediction (see Appendix A). The ROC curves were computed for each model and some comparisons were made using the De Long et al. procedure [24].

Calibration of models was computed distributing the observed cases into quintile classes of the estimated probabilities. To compare the performance of the various models we used the chi-squared test and a semi-quantitative procedure consisting in the calculation of the first order moment of each distribution (i.e., the baricentrum).

Finally, we produced Cox models with CHD and separately with CVD as end-points, using only 3 classic CHD risk factors (cigarette smoking, systolic blood pressure, serum cholesterol) plus age and separately the 6 risk factors adopted by the IDF-MS definition plus age.

## 3. Results

Mean levels of risk factors (Table 1) show that they exceed the cut-off limits for the IDF-MS definition in the case of systolic blood pressure, diastolic blood pressure and triglycerides. The prevalence of the IDF-MS components was high for hypertension and rather low for glucose while the overall prevalence of MS was 9.9%.

The correlation matrix of the seven risk factors (not reported in detail) provided low levels of the linear correlation coefficients (R), with the only exception of relatively high values for triglycerides versus total serum cholesterol (positive) and versus HDL cholesterol (negative), explaining around 10% of variance, a finding that contradicts the claimed high association across the MS risk factors. Curiously enough, the two highest correlation coefficients included a variable (total serum cholesterol) unfortunately omitted by the official MS definition for IDF.

The twelve Cox models (six without and six with the inclusion of total serum cholesterol) are reported in Table 2, Table 3, Table 4 and Table 5, but by themselves they do not explain the problem. In Table 2, which deals with models for CVD fatal events without total serum cholesterol, the MSLINEAR solution shows that not all components and risk factors are associated with significant coefficients and only those of systolic blood pressure and glucose were statistically significant. Moreover, a comparison of the IDF-MS model with the MSLINEAR model using the AIC suggests that the model using the original units of measurement of the risk factors provided a better outcome. Similar findings are reported in the parallel models also including total serum cholesterol (Table 3). Again, it was clear that a model using the original levels of risk factors produced better predictions. In other words, a traditional Cox model using the same risk factors provided a better prediction of events than employing arbitrary classes and combinations of categorized risk factors (clusters) as suggested by the IDF-MS criteria. The performance of the MSFA model was in between the other two, worse than the MSLINEAR one, but better than the MSIDF. Beyond that, the models including total serum cholesterol (Table 3) showed better predictions than the correspondent ones solved without total serum cholesterol (Table 2) after having computed AIC in parallel couples of models. The coefficient of total serum cholesterol was always significant although cases of CHD, that are those surely related to cholesterol [15], are only 48% of total CVD cases.

Parallel findings are presented in Table 4 and Table 5 where the end-point is CHD mortality. They replicated those presented for CVD, with the linear models performing better than those based on the IDF criteria. The only substantial difference being the different performance of the MSFA model that, in this case, was not better than that of MSIDF.

Using the AIC, we compared models including with those not including total serum cholesterol, which showed that the former ones out-performed the latter ones in all cases (details not reported).

Findings derived from the above-mentioned Cox models were compared for their performance with the ROC-AUC curves and calibration tests (Table 6, Table 7 and Table 8). All ROC, across the various models, were statistically significant but models using the linear approach were larger and significantly different from the others. Something similar happened with the calibration procedures where for all models the chi-square statistics were significant but larger levels of the first order moment (or baricentrum) of the distributions were found for the linear models.

In order to give additional evidence on the weakness of the IDF-MS approach, we produced another simple analysis computing Cox models with CHD and CVD mortality, separately, as the end-point, using age, smoking habits, systolic blood pressure and total serum cholesterol (the three standard, classical CHD risk factors) plus age. These two models (Table 9: see also Appendix A) were compared with the linear models for CVD (Table 2) and for CHD (Table 4) using the AIC on loglikelihood statistics evaluating the performance of the two types of models. Findings showed a large advantage for adopting the old and standard set of three risk factors for CHD and similarly for CVD as the end-point, over the MSLINEAR model.

## 4. Discussion

The purpose of this analysis was to explore whether the individual analytical handling of the risk factors produced (or not) better predictive models of CVD and/or CHD fatal events compared with the procedure proposing to cluster them into the IDF-MS approach [8]. This analysis showed for the first time, based on objective statistics, that the group of risk factors belonging to one of the current MS definitions produced more valuable predictive models when adopting their basic values and units of measurement rather than creating categorical classes by clustering them together. Beyond any other consideration, those categorial classes have an extreme variability of proposals, there are subclasses in the four risk factor groups and there is a frequent use of the item “or” when defining IDF-MS [8]. All this tends to complicate the correct interpretation of the conclusion stating that MS is either present or absent.

The Cox proportional hazards models predicting CVD/CHD mortality were compared using the AIC and clearly showed the superiority of the traditional analysis: there are therefore no advantages in selecting a group of few risk factors, giving them a name such as MS and treating them using ambiguous and a priori defined cut-off limits. It is also of interest that the MSFA models, created by clustering the risk factors following an a posteriori procedure, performed better than the MSIDF models created by clustering risk factors on the basis of “clinical opinions”, a clearly subjective judgment.

Following the official IDF-MS definition, its prevalence was about 10% in this study, which might be due to the relative rarity of BMI ≥ 30 units and whose presence in that definition is a “must”. However, even within a single scheme, the real possible combinations are enormous considering that a single component, for example, hypertension in the IDF proposal, includes four possibilities (i.e., no high blood pressure, high systolic only, high diastolic only, both high systolic and diastolic). Nevertheless, “hypertension” was the most common (72.1%) and at the same time the only component that was always significantly associated with events when treated independently, and even when separated into systolic and diastolic blood pressures. This high prevalence was clearly due to the choice of very low cut-off limits to define hypertension (that is deliberately a highly sensitive choice) contrasting the “clinical opinion” of about 50–60 year ago when hypertension was defined by the presence of systolic blood pressure above 180 mmHg. Incidentally, in our analysis, when systolic and diastolic blood pressures were fed into the same model there were no multicollinearity problems since tolerance was always high.

The dyslipidemic component of MS included HDL cholesterol that had a significant predictive role when treated independently, while the role of triglycerides, BMI and blood glucose, as predictors of cardiovascular events, was not so clear nor strong. Despite the existence of reports showing the direct and significant role of triglycerides in the prediction of CHD and CVD events [25,26] their importance seems still debated and uncertain [15]. Also, in our experience with three rather different Italian population studies significant contributions of triglycerides to CHD and CVD prediction were not disclosed [27,28,29]. In dedicated models from the present study with triglycerides as a unique covariate, the coefficients *p* values were 0.0046 and 0.0204 for CHD and CVD, respectively, suggesting serious interferences when put together with the other risk factors.

The predictive role of BMI in this analysis was practically null, but this was not a surprise on the basis of our experience with other Italian population studies. In fact, we found significant coefficients of BMI only when, in very long-term follow-ups, this variable was treated as a parabolic function and the end-point was age at death in cohorts practically extinct, still accompanied by a large number of other covariates [30]. The marginal predictive power of BMI and adiposity in the presence of other major risk factors has been largely confirmed [31]. In dedicated models from the present study with BMI as a unique covariate, the coefficients *p* values were 0.0038 and 0.0043 for CHD and CVD, respectively, again suggesting interferences with the other variables.

Also, blood glucose did not provide significant coefficients but in dedicated models where it was the unique covariate the coefficient *p* values were <0.0001 for both CHD and CVD as dependent variables. This simply says that the relatively small correlation of blood glucose with the other risk factors interferes with the production of a predictive role in multivariate analysis. This effect was already seen in other studies of our research group [28,29].

In this material, the high correlations across the various MS components were not found as claimed by the MS scholars, but they were large enough to interfere with the predictive power of other risk factors as outlined above. This is in part the reason why we ran a parallel series of models including also total serum cholesterol which showed to be systematically more predictive than the individual MS components as proposed by IDF. This is not a discovery since the relationship of total serum cholesterol (even better non-HDL cholesterol or LDL-cholesterol) with the CHD component of CVD mortality is a universal and recognized fact [15] but disregarded by the MS scholars [1,2,3,4,5,6,7,8,9,10,11] and their followers [12,13,14].

Any simple computer program, constructed adopting one of the many multivariate models available in the literature, will be more reliable and helpful than using one of the many and contrasting definitions of MS. In fact, by these means it is possible to identify cut-off limits of estimated probabilities covering clearly defined proportions of expected events.

Our analysis has limits due to the relatively small number of fatal CVD (*n* = 412) and CHD (*n* = 196) events, the forced use of BMI ≥ 30 units for the obesity component, the availability of only male subjects (as women had even fewer events), the use of mortality instead of incidence of CVD/CHD events and the population samples from a single country. However, if these limits were detrimental to accepting our conclusion, we should also negate a great deal of our current knowledge based on these shared defects [15]. There is no reason why the limits of the present analysis should have impaired the technical purpose of this analysis, potentially applicable to different problems outside the specific one of MS. However, due to the objective limits of this analysis the next step in this area should be a replica of the same analysis based on a different population, including—separately—men and women, with the availability of waist circumference, characterized by a larger proportion of events, compared with the present analysis and forcing total serum cholesterol in the system (or better non-HDL cholesterol, or LDL cholesterol). Similar conclusions may lead to a dismissal or systematical review of the MS principles.

It should also be recalled that the literature provides a large number of cardiovascular predictive scores, based on large population studies and properly tested. An example is the Euro-Score [32] that has had a great success being applied to many settings and different countries and no MS is defined there. Similarly, we do not present here “opinions”, that is a priori beliefs, but rather sound and numerically documented proof, among men recruited from residential cohorts, on the superiority of traditional multivariate analysis on covariates entered continuously to predict outcomes. The opposite end are the “opinions” expressed by Scientific Societies, Committees, Expert Groups or Editorialists, deciding what is what and how it should be valued [15,16], independent of any statistical evidence. Another analysis that has some similarities with ours, explored nine different criteria for the definition of MS and found that high levels of glucose, triglycerides and blood pressure were singularly associated with CVD events, which was not the case for waist circumference and HDL cholesterol [33].

We did not compare our findings with those from analyses dedicated to MS, facing the difficulty of selecting universally recognized references among the 100,000 available in the current literature. We did not enter the discussion on whether other (and which other) risk factors should be considered [1,2,3,4,5,6,7,8,9,10,11,12,13,14]. However, it is clear that by adding and adding more personal characteristics the consequently inflated “syndrome” will be able to predict all kinds of disease, all-cause mortality and age at death. Incidentally, our research group already developed this experiment using large numbers of risk factors and personal characteristics to predict age at death in extinct cohorts, without claiming to have discovered a new syndrome [30]. However, the exclusion of total serum cholesterol (or better non-HDL or LDL-cholesterol) seems to be a great mistake mainly in the face of the alternative decision in some proposals, which is to include C-reactive protein, inflammation indicators, homocysteine, etc., that probably are not causes but only consequent pathophysiological reactions to real causes.

## 5. Conclusions

**Figure 1:**
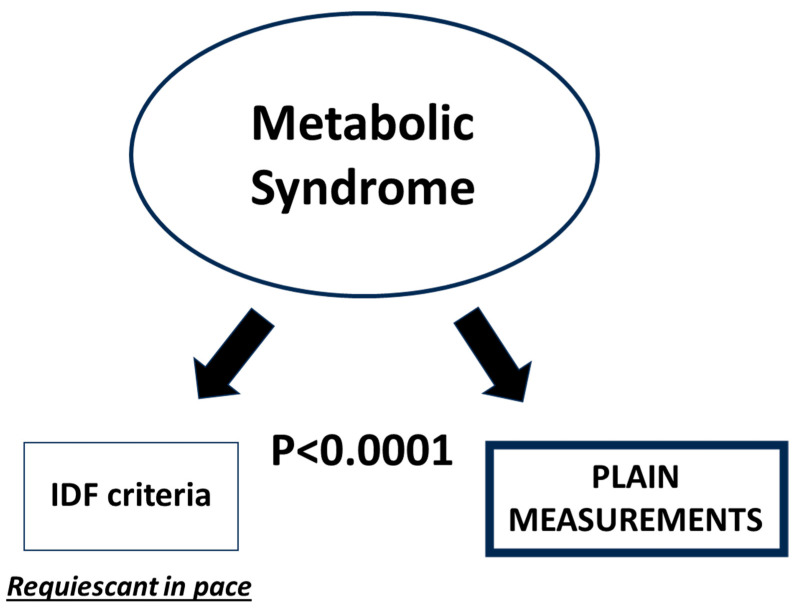
The results of this study clearly indicate that the originator of the Metabolic Syndrome idea and concepts was fully right when contrasting his creature [17,18] and indeed the IDF-MS criteria [8] should indeed rest in peace (R.I.P. = requiescant in pace in Latin) when compared with the individual components as risk factors treated by standard statistics for predictive purposes of CHD/CVD at least among apparently healthy men at enrolment from Italy, as explored in our study.

It seems that the spread of the MS concept is a kind of revenge of the “clinical opinion” thinking that in fact disregards and dismisses the accomplishments of populations studies that instead are based on the handling of statistically solid data. Under these circumstances the idea of MS is a kind of fiction, simply pushing to factual rethinking on how best to present and interpret results related to CVD/CHD risks. Our analysis has shown the superiority, in terms of predictivity, of models whereby the individual levels of risk factors were used continuously versus adopting a priori scores derived from cut-off limits and various combinations used in the MS approach. Thus, at least among apparently healthy men from residential cohorts from Italy, the Reaven conclusions for MS to rest in peace [17,18] do apply (Figure 1). CVD/CHD mortality predictions and MS are rather foes than friends and an essential conclusion from the present analysis is that for CHD prediction, total serum cholesterol omission is an error.

## Figures and Tables

**Table 1:** Baseline levels of risk factors and rates of MS components used in the analysis among 25,271 cardiovascular-disease-free men aged 35–70 years of the Italian RIFLE Study.

	Mean	Standard Deviation	Median	25th Percentile	75th Percentile
Body mass index, kg/m^2^	26.5	3.4	26.3	24.3	28.4
Systolic blood pressure, mmHg	136.9	20.0	135	121	150
Diastolic blood pressure, mmHg	86.2	11.2	85	80	91
HDL cholesterol, mg/dL	49.3	13.5	48	40	56
Triglycerides, mg/dL	150.3	107.0	123	90	178
Glucose, mg/dL	95.5	23.3	92	84	100
Total cholesterol, mg/dL	222.3	46.1	220	191	250
**Rates of IDF-MS components**					
		**Rate, %**		**Standard error**	
1. Obesity: Body mass index ≥ 30 units		14.8		0.2	
2. Hypertension: SBP ≥ 130 or DBP ≥ 85 mm Hg		72.1		0.3	
3. Dyslipidemia: HDL < 40 mg/dL or Triglycerides ≥ 150 mg/dL		45.6		0.3	
4. Glucose: >100 mg/dL		24.4		0.3	
**Rates of Analytical groups**					
		**Rate, %**		**Standard error**	
MSIDF = BMI ≥ 30 plus 2 or more components (¥)		9.9%		0.2	
MSFA (factor score of Factor Analysis)		All subjects involved			
MSLinear		All subjects involved		---	

(¥) This analytical group corresponds to the official definition of MS for the IDF.

**Table 2:** Three Cox models for prediction of all CVD not including serum cholesterol and Akaike Information Criterium. Units of measurement of variables as from Table 1.

Cox Models					
	Coefficient	*p*-Value	Delta	HR	95% CI
**Model MSIDF**					
Age	0.0903	<0.0001	9	2.25	2.01–2.53
MSIDF	0.4460	0.0008	1	1.56	1.20–2.03
**Model MSFA**					
Age	0.0878	<0.0001	9	2.20	1.96–2.48
MSFA	−0.2360	<0.0001	1	0.79	0.73–0.85
**Model MSLINEAR**					
Age	0.0770	<0.0001	9	2.00	1.77–2.26
BMI	−0.0154	0.3058	3.5	0.95	0.85–1.05
Systolic blood pressure	0.0141	<0.0001	20	1.33	1.18–1.49
Diastolic blood pressure	0.0092	0.1066	11	1.11	0.98–1.25
HDL cholesterol	−0.0046	0.2558	16	0.93	0.82–1.06
Triglycerides	0.0003	0.4935	110	1.03	0.94–1.13
Glucose	0.0060	<0.0001	23	1.15	1.08–1.22
**Akaike Information Criterion (AIC) from log-likelihood**					
	**Log-likelihood**	**AIC**	**Comparison of AICs**		
Model MSIDF	−3865.9	7735.8	MSLINEAR (*) versus MSIDF		
Model MSLINEAR	−3829.7	7667.4	MSLinear (*) versus MSFA		
Model MSFA	−3854.3	7704.7	MSIDF versus MSFA (*)		

Delta is the differential level of covariate for the computation of Hazard Ratio (HR). CI: confidence intervals. (*): model with better performance.

**Table 3:** Three Cox models for prediction of all CVD including serum cholesterol and Akaike Information Criterium. Units of measurement of variables as from Table 1.

Cox Models					
	Coefficient	*p*-Value	Delta	HR	95% CI
**Model MSIDF**					
Age	0.0902	<0.0001	9	2.85	2.01–2.53
Cholesterol	0.0034	0.0008	45	1.17	1.07–1.28
IDF	0.4074	0.0022	1	1.50	1.16–1.95
**Model MSFA**					
Age	0.0878	<0.0001	9	2.20	1.96–2.48
Cholesterol	0.0032	0.0021	45	1.15	1.05–1.27
FA score	−0.2256	<0.0001	1	0.80	0.74–0.86
**Model MSLINEAR**					
Age	0.0774	<0.0001	9	2.01	1.77–2.27
Cholesterol	0.0029	0.0104	45	1.14	1.03–1.26
BMI	−0.0178	0.2406	3.5	0.94	0.85–1.04
Systolic blood pressure	0.0139	<0.0001	20	1.32	1.17–1.49
Diastolic blood pressure	0.0087	0.1275	11	1.10	0.97–1.24
HDL cholesterol	−0.0076	0.0757	13	0.91	0.81–1.01
Triglycerides	−0.0002	0.7482	110	0.98	0.89–1.09
Glucose	0.0060	<0.0001	23	1.15	1.08–1.22
**Akaike Information Criterion (AIC) from log-likelihood**					
	**Log-likelihood**	**AIC**	**Comparison of AICs**		
Model MSIDF	−3860.5	7727.0	MSIDF versus MSLINEAR (*)		
Model MSLINEAR	−3823.5	7663.0	MSIDF versus MSFA (*)		
Model MDFA	−3849.8	7693.5	MSLINEAR (*) versus MSFA		

Delta is the differential level of the covariate for the computation of Hazard Ratio (HR). CI: confidence intervals. (*): model with better performance.

**Table 4:** Three Cox models for prediction of all CHD not including serum cholesterol and Akaike Information Criterium. Units of measurement of variables as from Table 1.

Cox Models					
	Coefficient	*p*-Value	Delta	HR	95% CI
**Model MSIDF**					
Age	0.0805	<0.0001	9	2.06	1.75–2.43
MSIDF	0.5660	0.0022	1	1.76	1.23–2.53
**Model MSFA**					
Age	0.0796	<0.0001	9	2.05	1.76–2.41
MSFA	−0.1721	0.0035	1	0.84	0.75–0.95
**Model MSLINEAR**					
Age	0.0669	<0.0001	9	1.83	1.53–2.18
BMI	0.0056	0.7960	3.5	1.02	0.88–1.18
Systolic blood pressure	0.0180	0.0044	20	1.43	1.21–1.70
Diastolic blood pressure	−0.0053	0.5193	11	0.94	0.79–1.13
HDL cholesterol	−0.0129	0.0354	13	0.85	0.17–0.99
Triglycerides	0.0006	0.2288	110	1.07	0.96–1.20
Glucose	0.0041	0.0651	23	1.10	0.99–1.22
**Akaike Information Criterion (AIC) from log-likelihood**					
	**Log-likelihood**	**AIC**	**Comparison of AICs**		
Model MSIDF	−1855.1	3714.2	MSIDF versus MSLINEAR (*)		
Model MSLINEAR	−1839.0	3690.0	MSIDF (*) versus MSFA		
Model MSFA	−1848.9	3714.4	MSLINEAR (*) versus MSFA		

Delta is the differential level of covariate for the computation of Hazard Ratio (HR). CI: confidence intervals. (*): model with better performance.

**Table 5:** Three Cox models for prediction of all CHD including serum cholesterol and Akaike Information Criterium. Units of measurement of variables as from Table 1.

Cox Models					
	Coefficient	*p*-Value	Delta	HR	95% CI
**Model MSIDF**					
Age	0.0802	<0.0001	9	2.06	1.75–2.43
Cholesterol	0.0053	0.0002	45	1.27	1.12–1.44
IDF	0.5020	0.0070	1	1.65	1.15–2.38
**Model MSFA**					
Age	0.0794	<0.0001	9	2.04	1.73–2.41
Cholesterol	0.0053	0.0035	45	1.27	1.12–1.44
FA score	−0.1525	0.0100	1	0.86	0.76–0.96
**Model MSLINEAR**					
Age	0.0676	<0.0001	9	1.84	1.54–2.19
Cholesterol	0.0054	0.0007	45	1.28	1.11–1.47
BMI	0.0014	0.9484	3.5	1.00	0.86–1.17
Systolic blood pressure	0.0177	0.0001	20	1.42	1.20–1.69
Diastolic blood pressure	−0.0062	0.4515	11	0.93	0.78–1.12
HDL cholesterol	−0.0187	0.0043	13	0.78	0.66–0.93
Triglycerides	−0.0002	0.8049	110	0.98	0.86–1.13
Glucose	0.0042	0.0632	23	1.10	0.99–1.22
**Akaike Information Criterion (AIC) from log-likelihood**					
	**Log-likelihood**	**AIC**	**Comparison of AICs**		
Model MSIDF	−1848.8	3703.6	MSIDF versus MSLINEAR (*)		
Model MSLINEAR	−1833.5	3681.0	MSIDF (*) versus MSFA		
Model MSFA	−1848.9	3703.8	MSLINEAR (*) versus MSFA		

Delta is the differential level of covariate for the computation of Hazard Ratio (HR). CI: confidence intervals. (*): model with better performance.

**Table 6:** ROC curves of 6 Cox models for CVD and comparisons following De Long et al. [24].

CVD				
Model	ROC	*p*-Value	Sensitivity	Specificity
MSIDF	0.704	<0.0001	75.5	55.5
MSFA	0.712	<0.001	72.8	60.9
MSLINEAR	0.732	<0.0001	76.5	60.0
MSIDF-cholesterol	0.709	<0.0001	81.6	52.1
MSFA-cholesterol	0.706	<0.001	73.3	61.4
MSLINEAR-cholesterol	0.736	<0.0001	75.5	62.0
**Comparisons**				
**Models without Cholesterol**			**Model with Cholesterol**	
	***p***			***p***
MSFA vs. MSIDF	0.1393		MSIDF vs. MSFA	0.1839
MSLINEAR vs. MSIDF	<0.0001		MSLINEAR vs. MSIDF	<0.0001
MSLINEAR vs. MSFA	0.0006		MSLINEAR vs. MSFA	0.0003

In the comparisons section the first model of each pair is the one with higher ROC level.

**Table 7:** ROC curves of 6 Cox models for CHD and comparisons following De Long et al. [24].

CHD				
Model	ROC	*p*-Value	Sensitivity	Specificity
MSIDF	0.690	<0.001	67.3	61.4
MSFA	0.689	<0.001	72.4	58.9
MSLINEAR	0.713	<0.001	50.6	55.4
MSIDF-cholesterol	0.705	<0.001	76.0	57.8
MSFA-cholesterol	0.702	<0.001	74.5	58.6
MSLINEAR-cholesterol	0.727	<0.001	84.2	52.0
**Comparisons**				
**Models without Cholesterol**			**Model with Cholesterol**	
	***p-*** **Value**			***p-*** **Value**
MSIDF vs. MSFA	0.8311		MSIDF vs. MSFA	0.6494
MSLINEAR vs. MSIDF	0.0194		MSLINEAR vs. MSIDF	0.0146
MSLINEAR vs. MSFA	0.0113		MSLINEAR vs. MSFA	0.0059

In the comparisons section the first model of each pair is the one with higher ROC level.

**Table 8:** Calibration of 6 Cox models for CVD and 6 models for CHD with number of events in quintiles of estimated risk.

	Quintiles						
Model	CVD						
	1	2	3	4	5	*p* of chi^2^	1st Order Moment
MSIDF	15	38	79	98	182	<0.0001	128.18
MSFA	16	30	66	123	177	<0.0001	130.60
MSLINEAR	14	29	60	121	188	<0.0001	134.09
MSIDF-cholesterol	14	35	78	106	179	<0.0001	128.74
MSFA-cholesterol	14	30	66	128	174	<0.0001	130.59
MSLINEAR-cholesterol	15	22	61	127	187	<0.0001	135.15
**Model**	**CHD**						
MSIDF	8	21	35	54	78	<0.0001	58.89
MSFA	9	18	33	58	78	<0.0001	59.60
MSLINEAR	10	15	26	59	86	<0.0001	62.37
MSIDF-cholesterol	6	19	31	57	83	<0.0001	58.09
MSFA-cholesterol	7	18	31	60	80	<0.0001	60.73
MSLINEAR-cholesterol	7	15	29	56	89	<0.0001	63.28

The 1st order moment is the baricentrum of the distribution.

**Table 9:** Cox models with CHD (196 cases) and CVD (412 cases) as end-points, using as predictors 3 classic risk factors. Units of measurement of variables as from Table 1.

Risk Factor	Delta	Coefficient	*p*-Value	HR	95% CI	Coefficient	*p* Value	HR	95% CI
		End Point CHD (a)				End Point CVD (b)			
Age	9	0.0726	<0.0001	1.92	1.61–2.29	0.0799	<0.0001	2.05	1.81–2.32
Cigarettes	11	0.0210	0.0004	1.26	1.23–1.57	0.0195	<0.0001	1.24	1.13–1.36
Systolic BP	20	0.0164	<0.0001	1.39	1.23–1.57	0.0182	<0.0001	1.44	1.33–1.56
Cholesterol	45	0.0046	0.0016	1.23	1.08–1.40	0.0026	0.0139	1.12	1.02–1.23

Delta is the differential level of covariate for the computation of Hazard Ratio (HR). CI = confidence intervals. Comparison of Akaike Criteria on log-likelihood statistics: (a) CHD: model age + 3 classic risk factors performs better than age + 6 MS risk factors of Table 4 and; (b) CVD: model age + 3 classic risk factors performs better than age+ 6 MS risk factors of Table 2.

## Data Availability

Data requisition requests to the RIFLE Research Group should be made by contacting Prof A. Menotti, Principal Investigator of the Project.

## Appendix A. Akaike Procedures

The Akaike procedures exploit the value of the log-likelihood, a metric that indicates the predictive power of multivariate models. The log-likelihood depends on the dependent variable and on the number of independent variables of a model and by itself cannot be compared with other similar metrics.

The Akaike Information Criterium (AIC) standardizes the log-likelihood as follows:

Akaike Information Criterium = 2K − 2(log-likelihood)

where K = number of independent variables.

Comparing two models, the one with lower AIC performs better than the one with higher AIC.

## References

[1] Avogaro P., Crepaldi G., Enzi G., Tiengo A. (1967). Association of hyperlipidemia, diabetes mellitus and of moderate level of obesity. Acta Diabetol. Lat..

[2] Reaven G. (1988). Role of insulin resistance in human diabetes. Diabetes.

[3] Alberti K.G., Zimmet P.Z. (1998). Definition, diagnosis and classification of diabetes mellitus and its complications. Part 1: Diagnosis and classification of diabetes mellitus provisional report of a WHO consultation. Diabet. Med..

[4] WHO (1999). Definition, Diagnosis and Classification of Diabetes Mellitus and Its Complications: Report of a WHO Consultation. Part 1, Diagnosis and Classification of Diabetes Mellitus.

[5] Cleeman J.I. (2001). Executive Summary of The Third Report of the National Cholesterol Education Program (NCEP) expert panel on detection, evaluation and treatment of high blood cholesterol in adults (Adult Treatment Panel III). JAMA.

[6] European Group for the Study of Insulin Resistance (EGIR) (2002). Frequency of the WHO Metabolic Syndrome in European Cohorts, and an alternative definition of an insulin resistance syndrome. Diab. Metabolism..

[7] Bloomgarden Z.T. (2003). American Association of Clinical Endocrinologists (AACE) Consensus conference on the insulin resistance syndrome 25–26 August, 2002, Washington, DC. Diabetes Care.

[8] Zimmet P., Alberti K.G.M.M., Serrano-Rios M. (2005). A new International Diabetes Federation (IDF) worldwide definition of the metabolic syndrome: The rationale and the results. Rev. Esp. Cardiol..

[9] Scheen A., Luyckx F., Esser N., Paquot N. (2024). Don’t neglect metabolic syndrome. Rev. Med. Liege.

[10] Malik S., Wong N.D., Franklin S.S., Kamath T.V., L’Italien G.J., Pio J.R. (2004). Impact of the metabolic syndrome on mortality from coronary heart disease, cardiovascular disease, and all causes in United States adults. Circulation.

[11] Hu G., Qiao Q., Tuomilehto J., Balkau N., Borch-Johnsen K., Pyorala K., DECODE Study Group (2004). Prevalence of the metabolic syndrome and its relation to all cause and cardiovascular mortality in nondiabetic European men and women. Arch. Intern. Med..

[12] Zhan Z.Q., Chen Y.Z., Huang Z.M., Luo Y.H., Zeng J.J., Wang Y., Tan J., Chen Y.X., Fang J.Y. (2024). Metabolic syndrome, its components, and gastrointestinal cancer risk: A meta-analysis of 31 prospective cohorts and Mendelian randomization study. J. Gastroenterol. Hepatol..

[13] Li M., Cao S.M., Dimou L., Wu L., Li J.B., Yang J. (2024). Association of metabolic syndrome with risk of lung cancer: A population-based prospective cohort study. Chest.

[14] Sebastian S.A., Padda I., Johal G. (2024). Cardiovascular-kidney-metabolic (CKM) syndrome: A state-of-the-art review. Curr. Probl. Cardiol..

[15] Visseren F.L.J., Mach F., Smulders Y.M., Carballo D., Koskinas K.C., Bäck M., Benetos A., Biffi A., Boavida J.-M., Capodanno D. (2022). 2021 ESC Guidelines on cardiovascular disease prevention in clinical practice developed by the Task Force for cardiovascular disease prevention in clinical practice with representatives of the European Society of Cardiology and 12 medical societies with the special contribution of the European Association of Preventive Cardiology (EAPC). Eur. J. Prev. Cardiol..

[16] De Geest B., Mishra M. (2023). The metabolic syndrome in obese and non-obese subjects: A reappraisal of the syndrome X of Reaven. Eur. J. Prev. Cardiol..

[17] Reaven G.M. (2005). The metabolic syndrome: Requiescat in pace. Clin. Chem..

[18] Reaven G.M. (2005). Just being alive is not good enough. Clin. Chem..

[19] Kahn R., Buse J., Ferrannini E., Stern M. (2005). The metabolic syndrome: Time for a critical appraisal. Joint statement from the American Diabetes Association and the European Association for the Study of Diabetes. Diabetes Care.

[20] Menotti A., Lanti M. (2007). The metabolic syndrome, the Guy syndrome and the hypofit syndrome. J. Cardiovasc. Med..

[21] The RIFLE Research Group (1993). Presentation of the RIFLE Project. Risk factors and Life Expectancy. Eur. J. Epidemiol..

[22] World Health Organization (1975). International Classification of Diseases and Causes of Death.

[23] Akaike H., Petrov B.N., Csáki F. (1973). Information theory and an extension of the maximum likelihood principle. 2nd International Symposium on Information Theory, Tsahkadsor, Armenia, 2–8 September 1971.

[24] De Long E.R., De Long P.M., Clarke-Pearson D.L. (1988). Comparing the areas under two or more correlated receiver operating characteristic curve: A non-parametric approach. Biometrics.

[25] Hokanson J.E., Melissa Austin M.A. (1996). Plasma triglyceride level is a risk factor for cardiovascular disease independent of high-density lipoprotein cholesterol level: A metaanalysis of population-based prospective studies. J. Cardiovasc. Risk.

[26] Kirby M. (2021). Lipids and lipid-modifying therapy. Men’s Health.

[27] Menotti A., Scanga M., Morisi G. (1994). Serum triglycerides in the prediction of coronary artery disease. An. Italian experience. Am. J. Cardiol..

[28] Menotti A., Lanti M., Zanchetti A., Botta G., Laurenzi M., Terradura-Vagnarelli O., Mancini M. (2011). The role of HDL cholesterol in metabolic syndrome predicting cardiovascular events. The Gubbio population Study. Nutr. Metab. Cardiovasc. Dis..

[29] Menotti A., Puddu P.E. (2019). Epidemiology of heart disease of uncertain etiology: A population study and review of the problem. Medicina.

[30] Menotti A., Puddu P.E., Catasta G. (2022). Determinants of longevity and age at death in a practically extinct cohort of middle-aged men followed-up for 61 years. Aging Clin. Exp. Res..

[31] Wormser D., Kaptoge S., Di Angelantonio E., Wood A.M., Pennells L., Thompson A., Sarwar N., Kizer J.R., Lawlor D.A., Emerging Risk Factors Collaboration (2011). Separate and combined associations of body-mass index and abdominal adiposity with cardiovascular disease: Collaborative analysis of 58 prospective studies. Lancet.

[32] Conroy R.M., Pyörälä K., Fitzgerald A.P., Sans S., Menotti A., De Backer G., De Bacquer D., Ducimetière P., Jousilahti P., Keil U. (2003). Estimation of ten-year risk of fatal cardiovascular diseases in Europe: The SCORE project. Eur. Heart J..

[33] Vera-Ponce V.J., Zuzunaga-Montoya F.E., Romero L.E.M.V., Loayza-Castro J.A., Manrique E.J.O., Valladares-Garrido M.J., Vigil-Ventura E., Tapia-Limonchi R. (2024). Evaluation of nine forms of Metabolic Syndrome diagnosis as risk for cardiovascular disease: An analysis of isolated and combined metabolic factors. J. Endocrinol. Metab..

